# High quality genome assembly of the amitochondriate eukaryote *Monocercomonoides exilis*

**DOI:** 10.1099/mgen.0.000745

**Published:** 2021-12-24

**Authors:** Sebastian Cristian Treitli, Priscila Peña-Diaz, Paweł Hałakuc, Anna Karnkowska, Vladimír Hampl

**Affiliations:** ^1^​ Department of Parasitology, Faculty of Science, Charles University, BIOCEV, Průmyslová 595, 252 42 Vestec, Czech Republic; ^2^​ Institute of Evolutionary Biology, Faculty of Biology, Biological and Chemical Research Centre, University of Warsaw, Warsaw, Poland

**Keywords:** *Monocercomonoides*, amitochondriate, genome, nanopore

## Abstract

*Monocercomonoides exilis* is considered the first known eukaryote to completely lack mitochondria. This conclusion is based primarily on a genomic and transcriptomic study which failed to identify any mitochondrial hallmark proteins. However, the available genome assembly has limited contiguity and around 1.5 % of the genome sequence is represented by unknown bases. To improve the contiguity, we re-sequenced the genome and transcriptome of *M. exilis* using Oxford Nanopore Technology (ONT). The resulting draft genome is assembled in 101 contigs with an N50 value of 1.38 Mbp, almost 20 times higher than the previously published assembly. Using a newly generated ONT transcriptome, we further improve the gene prediction and add high quality untranslated region (UTR) annotations, in which we identify two putative polyadenylation signals present in the 3′UTR regions and characterise the Kozak sequence in the 5′UTR regions. All these improvements are reflected by higher BUSCO genome completeness values. Regardless of an overall more complete genome assembly without missing bases and a better gene prediction, we still failed to identify any mitochondrial hallmark genes, thus further supporting the hypothesis on the absence of mitochondrion.

## Data Summary

Raw DNA and RNA sequence reads are archived at NCBI Sequence Read Archive (SRA) under accession numbers SRR15678500-SRR15678502.

This Whole Genome Shotgun project has been deposited at DDBJ/ENA/GenBank under the accession LSRY00000000. The version described in this paper is version LSRY02000000.

**Impact Statement**

Inference of biological features from the genomic and transcriptomic data sets is a common and powerful approach that has significantly expanded our horizons. At the same time, the weight of the conclusions is always affected by the completeness of the data, which for the large and complex eukaryotic genomes rarely reaches 100 %. We have previously used genomic and transcriptomic data as an argument for a unique absence of mitochondrion in the flagellate *Monocercomonoides exilis*. Knowing that the assembly based on 454 sequencing technology is fragmented and contains gaps, we revisit the case with nowadays-available third-generation sequencing technologies. Our results confirm the amitochondrial status of *M. exilis* and provides a unique view of the complexity and organisation of the genome. The study demonstrates that third-generation sequencing technologies can provide significant improvements in contiguity. At the same time, it demonstrates the difficulty of transferring the annotations from the previous versions, which we have overcome by a very careful but complicated iterative procedure of our design. We argue that annotation transfer is an important step, which stores the previously obtained information, and should be used when possible.

## Introduction

Oxymonads (Preaxostyla, Metamonada) are flagellates inhabiting mainly the guts of wood-feeding insects with some species also found in the gut of vertebrates [1–4]. They are among the least studied groups of protists, mainly because very few of them can be cultured *in vitro*. This and the fact that all available cultures are polyxenic [3], pose a challenge to obtain high quality genomic data. Only recently, oxymonads entered the genomics era with the publication of the first oxymonad genome of *M. exilis* [5]. The study brought a remarkable finding by demonstrating the absence of any mitochondrial hallmark proteins, including those of the Iron-Sulphur Cluster (ISC) assembly pathway, an observation used as the main argument for a hypothesis that the organism has no remnant of mitochondrion [5]. The genomic draft of the second oxymonad, *Streblomastix strix*, was obtained from whole genome amplified DNA [6] of micromanipulated cells isolated from the gut of *Zootermopsis angusticollis*. Similarly to *M. exilis, S. strix* genome also lacked the mitochondrial ISC pathway and it was demonstrated that this pathway was substituted by the SUF pathway acquired by horizontal gene transfer already in the common ancestor of Preaxostyla [7]. Both oxymonad genome drafts provided valuable information about the biology of oxymonads and their metabolic capacities. However, in both cases the genome assemblies are fragmented. The genome of *S. strix* is assembled in more than fifty thousand scaffolds with an N50 value of approximately 5 kbp [6]. The situation is better in the case of *M. exilis*, where the genome is assembled in approximately 2000 scaffolds with an N50 of around 71 kbp [7]. Still, approximately 1.1 Mbp of data in the latter assembly are represented by unknown bases [7]. These may represent repetitive elements but at the same time, these could contain genes including the intensively searched mitochondrial markers which were simply not captured during sequencing. Improving the genome assembly for either oxymonad would allow us to support the hypothesis on its amitochondriality and to better understand genome structure and organization.

Long-read sequencing technologies like Oxford Nanopore Technologies (ONT) and Pacific Biosciences (PacBio), have recently been very helpful in improving genome assemblies of several model and non-model organisms [8–10]. ONT has been successfully used for generating draft genome assemblies of plants [11, 12], metazoans [13, 14], fungi [15] and even protists [9, 16], and it consistently produced much more contiguous assemblies. The main drawback of long-read sequencing remains the consensus accuracy, however, recent improvements in sequencing chemistry as well as base-calling algorithms for ONT improved this issue. Also, third-party tools have been developed to polish the assemblies either using long-reads [17, 18] or Illumina short reads [19] aiming to improve the overall accuracy of the sequences.

In this study we present a new highly contiguous genome assembly of *M. exilis* strain PA203 generated with the help of ONT sequencing data. We transferred the annotations from the previous published assembly to the new one while maintaining the locus tags and drastically reduced the number of incomplete gene models. With the help of a new version of ONT based transcriptome we further improved the gene predictions and added high quality UTR annotations, which allowed us to identify putative regulatory motifs in the UTR regions. We show that despite the larger size and higher contiguity of the assembly, mitochondrial hallmark proteins have not been detected supporting the hypothesis on the absence of mitochondrion.

## Methods

### DNA, RNA isolation and cDNA synthesis

*Monocercomonoides exilis* strain PA203 was cultured in a modified TYSGM media [20] as described previously [3]. Prior DNA isolation, 22 litres of culture were filtered as described previously [4, 5] to remove most of the bacterial contaminants. The filtered cells were collected at 1200 ***g*** for 10 min at 4 °C. The genomic DNA (gDNA) was isolated using the Qiagen MagAttract HMW DNA Kit (Qiagen) according to the manufacturer’s protocol.

Total RNA was isolated from 500 ml of culture, which was initially filtered through a filter paper to remove large bacterial aggregates followed by filtration through a 3 µm nylon filter, without washing with fresh media. The filtered cells were collected at 1200 ***g*** for 10 min at 4 °C and the total RNA was isolated using TRI reagent (Sigma-Aldrich). The mRNA was purified from total RNA using Dynabeads mRNA Purification Kit (Thermo Fisher Scientific). The cDNA was synthesized using the SMARTer PCR cDNA Synthesis Kit (Takara Bio Group) through 18 cycles of amplification.

### Library preparation and sequencing

For ONT gDNA sequencing, two libraries from 4 µg of gDNA each were prepared. The gDNA was sheared to ~20 kbp using Covaris g-TUBES (Covaris Ltd, UK). After shearing, the libraries were prepared using Ligation sequencing kit (SQK-LSK108) according to the manufacturer’s protocol. Each library was loaded onto an R9.4.1 Spot-On Flow cell (FLO-MIN106) and sequencing was performed for 48 h on a MinIon Mk1B machine using MinKNOW 2.0 software. For ONT transcriptome sequencing, 1 µg of cDNA was used. The library was prepared using Ligation sequencing kit (SQK-LSK109) according to the manufacturer’s protocol and loaded onto a R10.3 Spot-On Flow cell (FLO-MIN111). Sequencing was performed for 24 h on a MinIon Mk1B machine using MinKNOW 3.6.5 software. For Illumina genomic sequencing, one pair-end gDNA library was prepared using TruSeq DNA PCR free kit (Illumina, San Diego, CA) according to the manufacturer’s protocol and sequenced on Illumina MIseq PE 2×300 bp at the Genomic Core facility, Faculty of Science, Biocev, Czech Republic.

### Genome assembly polishing and decontamination

Base calling of the ONT reads for genomic and transcriptomic data was performed using Guppy 3.0.3. Adapters and chimeric reads were removed using Porechop v0.2.3 (https://github.com/rrwick/Porechop). The assembly of the genome was performed using Canu 1.8 [21] with corMinCoverage set to zero and corOutCoverage set to 100 000. Following assembly, the data was binned using the tetraESOM method [22] and the eukaryotic bin was checked for bacterial contamination using a combination of blastn and blastp as described previously [3]. The final eukaryotic genome assembly was polished using the ONT reads with Nanopolish [17] followed by polishing using the Illumina short reads with Pilon v1.21 [19]. A schematic overview of the entire workflow for genome assembly, annotation transfer and polishing is shown in Figs S1–S5 (available in the online version of this article).

### Annotation transfer and gene prediction polishing

Transfer of the previously published gene predictions [5, 7] to the new ONT assembly, was made in four semi-automatic steps. First, *de novo* gene model prediction was performed on the ONT assembly using Augustus 3.2.3 [23]. The predicted gene models were corrected using EVM [24], but instead of transcriptome input, we used the previously extracted coding sequences (CDS) from the original 454 genome assembly annotation which had been mapped to the ONT assembly with PASA [24] as ‘evidence’. After running EVM, we extracted the nucleotide and protein sequences of the newly predicted genes and clustered them with the sequences of the genes from the original 454 based prediction [5, 7], using cd-hit [25] with -s 1 and -c 1 (same length, 100 % identity). The clusters containing representatives from both genomes were removed, and these genes were considered transferred. The genes from the 454 assembly forming clusters without representatives from the ONT assembly were considered non-transferred and were used in the second step where they were transferred to the ONT assembly using RATT [26]. After RATT transfer, we extracted the CDS and protein sequences of the transferred genes and clustered them with the 454 based prediction using cd-hit as described above. Genes from 454 based prediction forming clusters without representatives from the ONT assembly, were used as input in the third step, in which we took the CDSes of these genes and mapped them to the ONT assembly with gmap [27]. After mapping, the nucleotide and protein sequences of the mapped genes were extracted and clustered with the 454 based prediction using cd-hit [25] with the parameters -c 0.95 s 1. The genes that failed to transfer even after this stage were mapped to ONT assembly using the PASA pipeline [24]. After mapping, protein sequences were predicted on the PASA output using Transdecoder and they were clustered with the 454 based prediction using cd-hit with -c 0.95 s 1. Finally, the genes which were not transferred in all previous steps were manually investigated, corrected, and transferred when possible. The procedure is summarized graphically in Fig. S1. The gene models from *de novo* prediction on the ONT assembly which did not overlap with the transferred predictions even after manual curation were added as new gene predictions.

After annotation transfer, prediction improvement and UTR annotation were performed using the ONT generated transcriptomic data and the PASA pipeline. First, the ONT generated cDNA reads were checked for chimaeras using Porechop. The non-chimeric reads were further classified into ‘full- length’ and ‘non-full-length’ reads using pychopper (https://github.com/nanoporetech/pychopper) and the adapters used for cDNA amplification were trimmed using Porechop.

To improve mapping to the genome, the classified and trimmed cDNA reads were corrected with the error correction module of Canu 1.8. and oriented according to the orientation of the transcript sequences taken from the transferred gene models. This step generated three individual subsets of reads: ‘oriented reads’, ‘unoriented reads’, and reads which do not overlap with any of the gene predictions present in our prediction file. Each of this subset was used as input into the PASA pipeline individually. First, the oriented reads were used as input into PASA with the parameters --transcribed_is_aligned_orient and --stringent_alignment_overlap set to 30.0. In the second stage, the unoriented reads were used as input into PASA with the parameter --stringent_alignment_overlap set to 30.0. In the third stage, the reads which did not overlap with any gene prediction were used as input into PASA as ‘full-length’ transcriptome input and with --stringent_alignment_overlap set to 30. In the final stage, the reads classified as ‘full-length’ by pychopper were used as input into PASA as ‘full-length’ transcriptome input and with --stringent_alignment_overlap set to 30. For all PASA runs, we used both blat and gmap as aligners and to validate the transcript alignments at least 60 % of the read length must have been aligned with at least 90 % identity.

After each PASA run, the gene prediction was updated using the annotationCompare module from PASA. For the first round, the comparison was made against the transferred gene models, but for subsequent runs the comparison was made against the manually curated output of the previous PASA annotationCompare run. Manual investigation after each PASA comparison focused on: (I) predictions whose protein sequence was modified, (II) predictions where the 3′UTR prediction was longer than 500 bp and (III) predictions where the 5′UTR prediction was longer than 100 bp. If the prediction was not in agreement with the transcriptomic support the gene models were modified.

Potential sequence errors in the ONT assembly were investigated based on observations during annotation transfer and annotation update with PASA. In cases of potential gene merging or splitting in disagreement with the transcriptome, we investigated the genome sequence to verify whether there were any mismatches/insertions/deletions uncorrected by Nanopolish or Pilon. For this we mapped back the Illumina genomic reads to the scaffolds using BWA aligner [28] and potential insertions/deletions were manually investigated in IGV [29].

### Genome completeness and motif analysis

The completeness of the ONT assembly was estimated using CEGMA [30]. BUSCO v3 with the eukaryota odb9 dataset was used to estimate the improvement of the prediction completeness after annotation transfer as well as after each annotation update using the ONT generated transcriptome.

To investigate the polyadenylation signals, we extracted only 3′UTR sequences that completely mapped to the ONT transcriptome reads classified as ‘full-length’ (see above) and their predicted 3′ end mapped right before the start of the oligodT primer used in reverse transcription. We considered this trait as a hallmark of a correctly predicted 3′UTR. The last 100 bp of the full length 3′UTR’s were extracted and motifs were searched in the UTR using STREME [31] from the MEME suite package. After the motif sequences were identified, their position in the sequence and their probability for each position was computed using Centrimo [32].

The Kozak consensus sequence was searched in a subset of 5′ UTR sequences from the manually curated genes [7]. This sorting would avoid artefacts due to incorrect start codon prediction. Ten base pairs upstream and downstream of the start codon were extracted and their consensus motifs were analysed using Weblogo [33].

### Search for putative mitochondrial proteins

The search for putative mitochondrial proteins was performed in a similar way as described previously [5]. Briefly, a custom mitochondrial protein sequence database was established using the MitoMiner v4.0 database [34]. The experimentally confirmed proteins (at least one GFP-tagging experiment or three different mass spectroscopy experiments) coming from *H. sapiens, M. musculus, R. norvegicus, D. rerio*, *S. cerevisiae* and *S. pombe* were used and supplemented by the published MROs’ protein sets from sixteen species [35–44]. Redundant homologues (90 % similarity threshold) were removed from the database using cd-hit [25]. The resulting non-redundant database contained 6979 proteins. Reciprocal blast analysis was performed for each set of data with an e-value threshold of 0.001. Hidden Markov Model (HMM) searches were used to identify proteins involved in protein import and translocation, as these were shown to be often divergent [42]. Searches were done in HMMER 3.1b2 [45] using HMMs profiles used in Karnkowska *et al.* 2016 [5].

Mitochondrial targeting signals were searched using TargetP v1.1 [46] and MitoFates v1.1 [47]. Proteins with probability of mitochondrial localisation >0.5 indicated by both programmes were considered for manual verification. To find tail-anchored proteins, transmembrane domains (TMDs) for all analysed proteins were predicted using TMHMM2.0 [48]. Proteins with TMD within 32 amino acids from C-terminus were kept for manual verification. The mitochondrial β-barrel outer membrane proteins (MBOMPs) search has been conducted using the pipeline described by Imai *et al.* 2011 [49]. The pipeline firstly identifies β-signal (P_o_xGh_y_xH_y_xH_y_ motif) in the C-terminus of protein required for the insertion into the membrane. Subsequently, the secondary structure of 300 amino acids preceding the β-signal is analysed using PSIPRED [50] to check for typical β-structure. Candidate sequences, with at least 25 % of β-strand, no more than 10 % of the α-helical structure and no more than 50 % of the eight residues of β-signal predicted as α-helical structure, were further analysed.

All candidate proteins encompassed in at least one of the methods described above were blasted against NCBI-nr and the best hit was kept, without 'low quality protein', 'hypothetical', 'predicted', 'unnamed', 'unknown', 'uncharacterized' in the description. For each protein, the Gene Ontology categories were assigned using InterProScan-5.36–75.0 [51]. All candidate proteins were combined with a NCBI-nr blast and InterProScan search results. Finally, each candidate protein was manually inspected for resemblance to known mitochondrial or mitosomal proteins. For most promising candidates, phylogenetic trees were reconstructed using IQ-TREE 1.6.12 [52] using default parameters.

## Results

### Genome assembly

ONT genome sequencing was performed using two 9.4.1 MinION flowcells. The two runs generated a total of approximately 12.9 Gbp of data with 3 097 486 base-called reads (N50=8.9 kbp) from which an assembly of 109.8 Mbp in 844 contigs was generated using Canu 1.8 [21]. After binning and decontamination, the consensus accuracy of the eukaryotic contigs was improved by polishing with Nanopolish [17] and ten rounds of Pilon [19]. The final *M. exilis* genome assembly consisted of 101 contigs with a total size of approximately 82.3 Mbp (Table 1) and a N50 value of 1 379 369 bp. This assembly will be referred as ONT assembly throughout the text. The previously sequenced draft genome of *M. exilis,* referred here as 454 assembly, was published in GiardiaDB (https://giardiadb.org/giardiadb/app/record/dataset/DS_3a6ccbfbcf). For the annotation transfer and comparisons described here we used the version 2019-07-27.

**Table 1.** General statistics of the previously published *Monocercomonoides exilis* 454 genome assembly and the ONT genome assembly obtained in this study

	454 assembly	ONT assembly
**Assembly size (bp**)	74 712 536	82 301 135
**G+C content (%**)	36.8	37.2
**No. of scaffolds/contigs**	2092/6648	101/101
**N50**	71 440	1 379 369
**No. of predicted protein coding genes**	16 767	18 152
**No. of partial gene models**	486	1
**Number of transferred genes/number of resulting gene models**	n/a	16448/16 323
**Number of non-transferred genes**	n/a	319
**Number of gene models fused/number of resulting gene models**	n/a	633/300
**Number of gene models split/number of resulting gene models**	n/a	54/110
**Gene models whose CDS was modified during transfer**	n/a	2838
**New gene models**	n/a	1829
**Mean gene length (bp**)	2704	2730
**Mean intergenic region length (bp**)	1484	1855
**Number of introns**	31 693	35 345
**Number of introns per gene**	1.90	1.95
**Mean intron length (bp**)	124	119
**Intron G+C content (%**)	25	27.6
**Number of genes with 3′ UTR**	6840	8354
**Mean 3'UTR length (bp**)	166	312
**Number of genes with 5′ UTR**	6967	5279
**Mean 5′ UTR length (bp**)	108	62

Evaluation using QUAST [53] revealed that 99.283 % of the 454 assembly is present in the new ONT assembly with a duplication ratio of 1.067 and only 50 scaffolds from 454 assembly failed to be identified. From these, 41 scaffolds were manually identified by blast and the remaining nine scaffolds (scaffold01565, scaffold01800, scaffold01857, scaffold01876, scaffold01882, scaffold01991, scaffold02045, scaffold02088, scaffold02141) are contaminants as they were mapped to binned prokaryotic sequences. Three of them (scaffold01876, scaffold01882, and scaffold01991) have been labelled as contaminants in a previous study [7]. The estimated completeness of the ONT assembly using CEGMA [30] is 67.34 %, exactly as for the published 454 assembly.

**Fig. 1.**
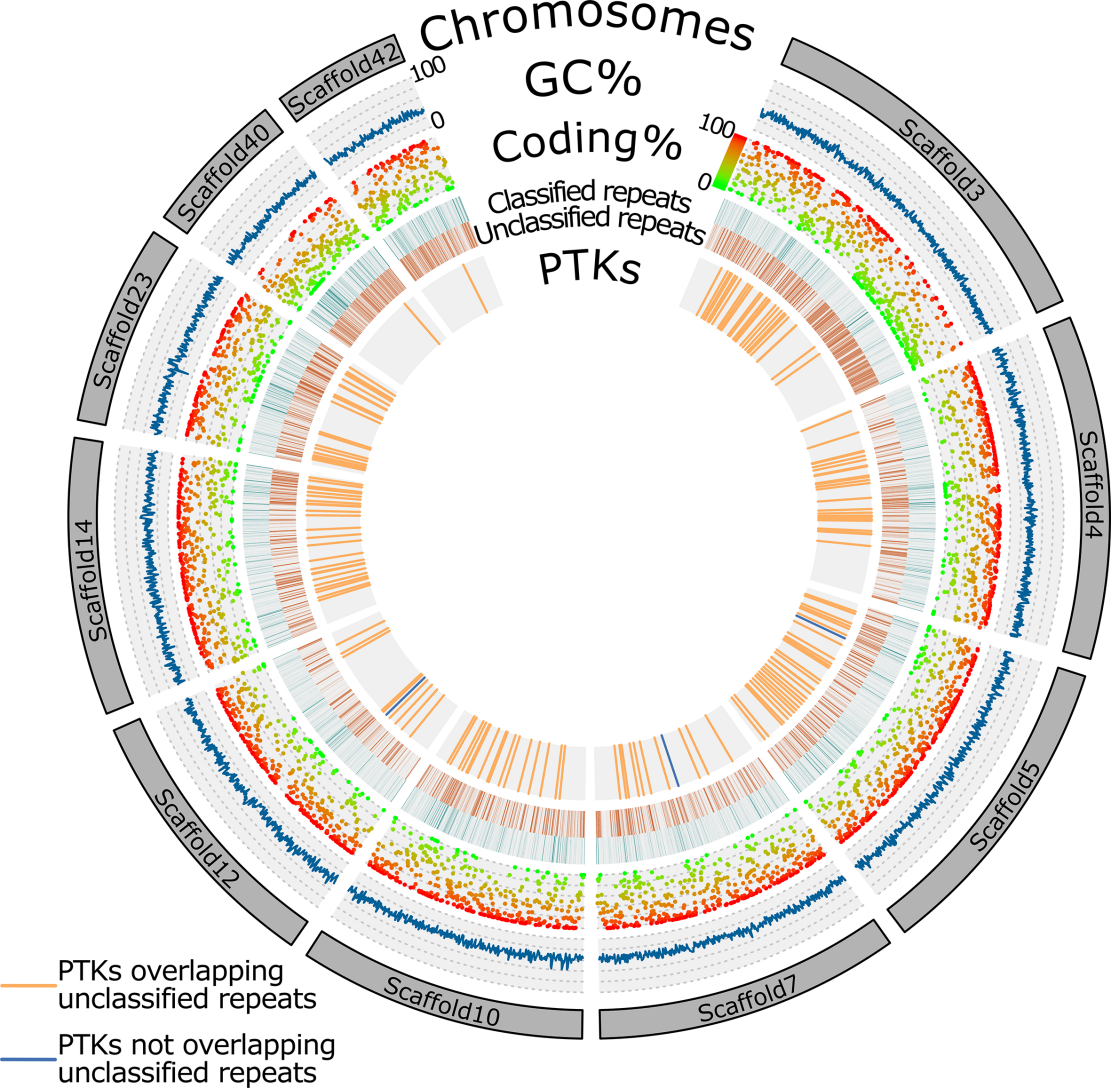
Circular representation of the ten complete chromosomes from the ONT assembly. The outermost track represents the chromosome-size scaffolds followed by GC content, coding percentage calculated for 5kbp windows, location and types of repetitive elements, and locations of protein tyrosine kinases (PTK) on the chromosomes. PTK’s overlapping unclassified repeats are represented by orange bars, and those not overlapping unclassified repeats are represented in blue.

The ONT assembly contained ten full-length chromosomes (with both ends capped by telomeric repeats) as well as 65 contigs bearing telomeric repeats at one of their ends. The size of the full-length chromosomes varied between 2.54 and 0.86 Mbp. Investigating their genomic organization revealed that on average 62.6 % of their length is covered by coding regions. The coding sequences tend to cluster together forming high density coding regions separated by regions with low coding density. This often correspond to areas with increased density of classified repeats (Fig. 1). This arrangement creates gene-dense regions where the gene models overlap with one another, particularly in the UTR regions.

### Annotation transfer

Before annotation transfer, we used RepeatModeler [54] and RepeatMasker to identify and mask repetitive elements. We identified approximately 37.8 Mbp of the ONT assembly as repetitive. Most of the repetitive elements were unclassified (~28.9 Mbp) and their distribution varied from chromosome to chromosome; some chromosomes (e.g. scaffold3, scaffold40, scaffold43) display higher density of unclassified repeats (Fig. 1). The classified repeats were far less abundant (Fig. 1) and were represented mainly by DNA transposons (3.72 Mbp), simple repeats (2.74 Mbp), LTR elements (1.41 Mbp) and low-complexity repeats (0.99 Mbp) (Table 2). We noticed that many of the unclassified repeats overlapped with various protein tyrosine kinases (Fig. 1). These kinases form one of the largest identified gene families in the genome of *M. exilis* [7]. For this reason, we masked only the classified repeats before *de novo* prediction on the ONT assembly.

**Table 2.** Repetitive elements identified in the ONT genome assembly of *M. exilis*

Type of repeats	No. masked bases (bp)	Percentage of the assembly
LTR elements	1 415 863	1.72
DNA transposons	3 722 012	4.52
Simple repeats	2 749 397	3.34
Low complexity	999 721	1.21
Unclassified	28 945 590	35.17
**Total**	37 832 583	45.97

The original 454-based assembly [5] contained 16 767 predicted gene models, of which 15 500 were transferred to the ONT genome assembly using a semi-automatic method (see Methods) and additional 948 were transferred manually. The 16 448 transferred gene models formed 16 323 gene models in the ONT assembly. Three hundred and nineteen gene models failed to be transferred (Table S1). These included gene models of poorly supported isoforms (149 models), gene models which would not make sense in the ONT assembly as they run in opposite direction of another corrected gene model (145 models), gene models present on scaffolds identified as contaminants (13 models) and gene models which were duplicated in the 454 assembly but not in the ONT assembly (12 models). During the manual transfer, we noticed that some gene models would need to be split or fused, as they disagreed with the transcriptomic data, causing either insertion of premature stop codons or long gene fusions. For this reason, we mapped back the Illumina reads on the assembled contigs and manually checked for any insertions, deletions, or mismatches uncorrected during Pilon polishing. We ended up manually correcting three scaffolds from the ONT genome assembly (scaffold33-1116896, scaffold80-53332 and scaffold89-33873). After completing the annotation transfer, we added 1660 new gene models, which were predicted on the ONT assembly but did not overlap with any of the previously transferred gene models, reaching the final number of 17 983 gene models.

### Prediction improvement with full-length ONT transcriptome

**Fig. 2.**
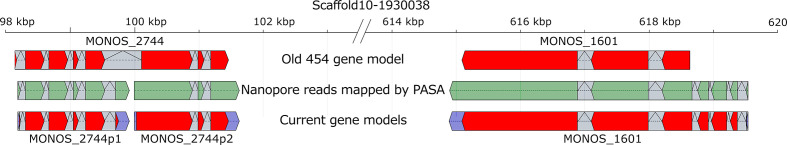
Two examples of gene prediction improvement on scaffold10. The first row represents the original 454 gene model. The second row represents full-length transcripts mapped to the genome using PASA. The last row represents the final gene models after prediction improvement with ONT generated transcriptome. Coding sequences are coloured in red, untranslated regions are represented in blue and introns are represented in grey.

In the next step, we used an ONT generated transcriptome to polish the predictions and add UTR annotations. Our ONT transcriptome sequencing generated 1.25 Gbp of sequences. We performed four independent runs of PASA, each of them addressing different issues with different parameters (see Methods). The mapped ONT transcriptome helped to improve the gene predictions either by extending the gene model ends, splitting the gene models, or fusing them. In the 454 assembly, many gene models were fused by addition of a long intron. One of these situations is represented in Fig. 2 using MONOS_2744 as example. The nanopore-generated transcripts clearly show that the model was incorrectly fused, and the mapped transcripts allowed PASA to automatically split the gene model in two parts. Similarly, mapped nanopore transcripts helped identify many incomplete gene models. Schematic representation of the gene model MONOS_1601 (Fig. 2) revealed that the mapped transcripts contained four more upstream exons, which were integrated in the final gene model. Excluding changes strictly related to the UTR sequences, the information from the ONT transcriptome led to the split of 42 gene models, fusion of 94, coding sequence update of 1157 models, and addition of 169 new gene models. We ended up with a final set of 18 152 gene models, 1829 of which were completely new.

Overall, from the transferred 454 assembly gene models, 2838 had their coding sequence modified, 633 were fused, and 54 were split during transfer and/or prediction improvement process (Table 1). Although we refer to the 1829 models as ‘completely new’, for most of them their DNA sequences were present in the 454 assembly, only 115 of them having less than 50 % of their sequence present in the 454 assembly. All previously predicted gene models retained their original locus tag names, with the exceptions of fused and split gene models whose naming highlights this trait. For example, locus tag MONOS_13233fu15373 indicates a fusion between MONOS_13233 and MONOS_15373, while locus tags MONOS_1266p1, MONOS_1266p2 indicate that these gene models are parts of the original gene model MONOS_1266.

**Fig. 3.**
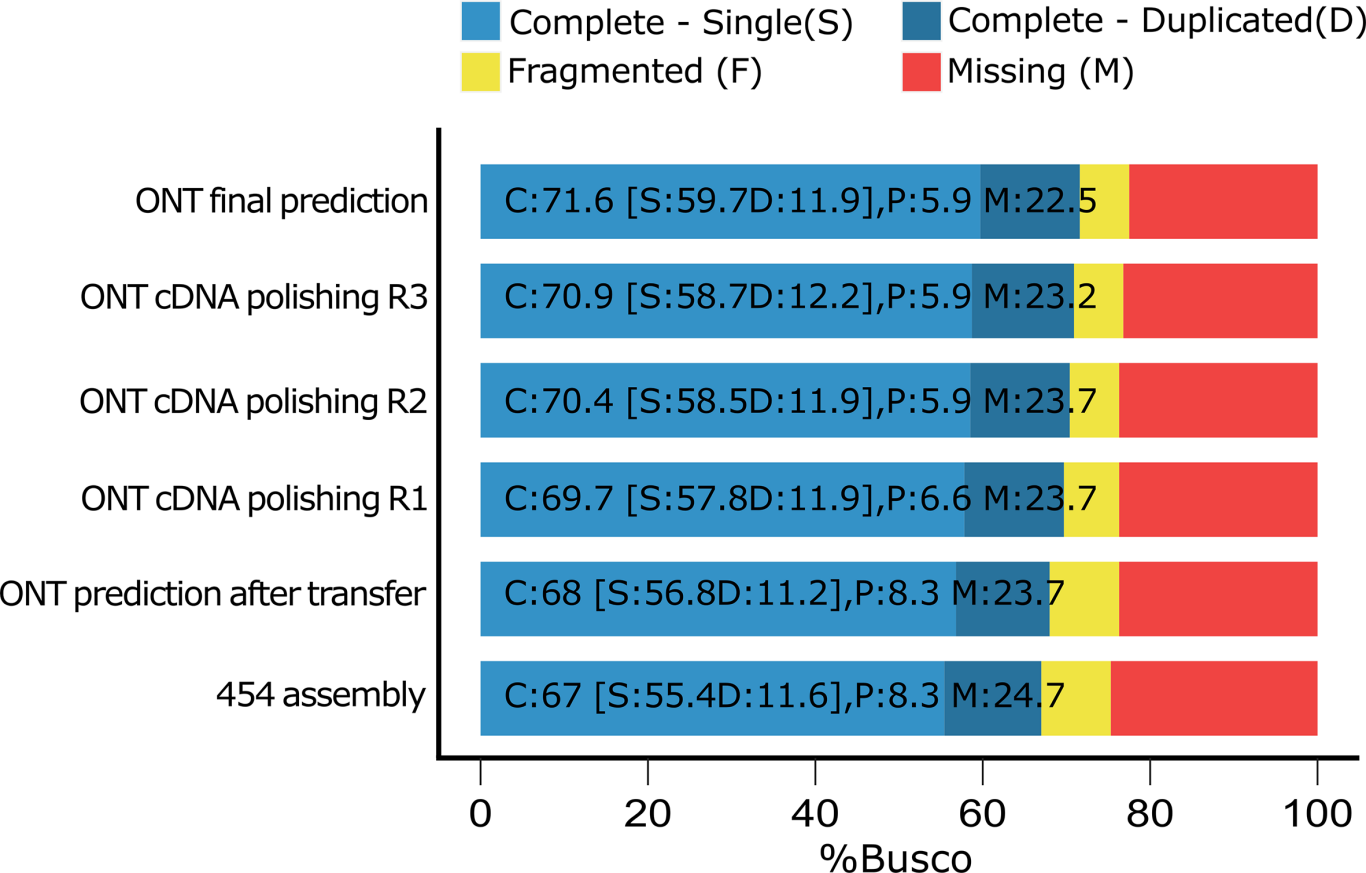
BUSCO genome completeness estimated on the list of predicted genes. The estimation was carried out using odbv9 dataset (*n*=303). The completeness was estimated after each step. ONT final prediction represents the published prediction after the fourth round of cDNA polishing.

To verify whether the use of ONT transcriptome had any positive effect on gene predictions we used BUSCO v3 [55] with the odbv9 dataset in protein mode to estimate the completeness of the gene predictions after each PASA step (Fig. 3). BUSCO displayed minor improvements in every step and the completeness of the final gene predictions was 71.4 %, i.e. more than 4 % higher than in the original assembly (67 %), while the percentage of partial BUSCOs decreased from 8.3 to 5.9 %, and the missing BUSCOs decreased by 2.3 % from 24.7 to 22.4 % (Fig. 3).

### UTR landscape

**Fig. 4.**
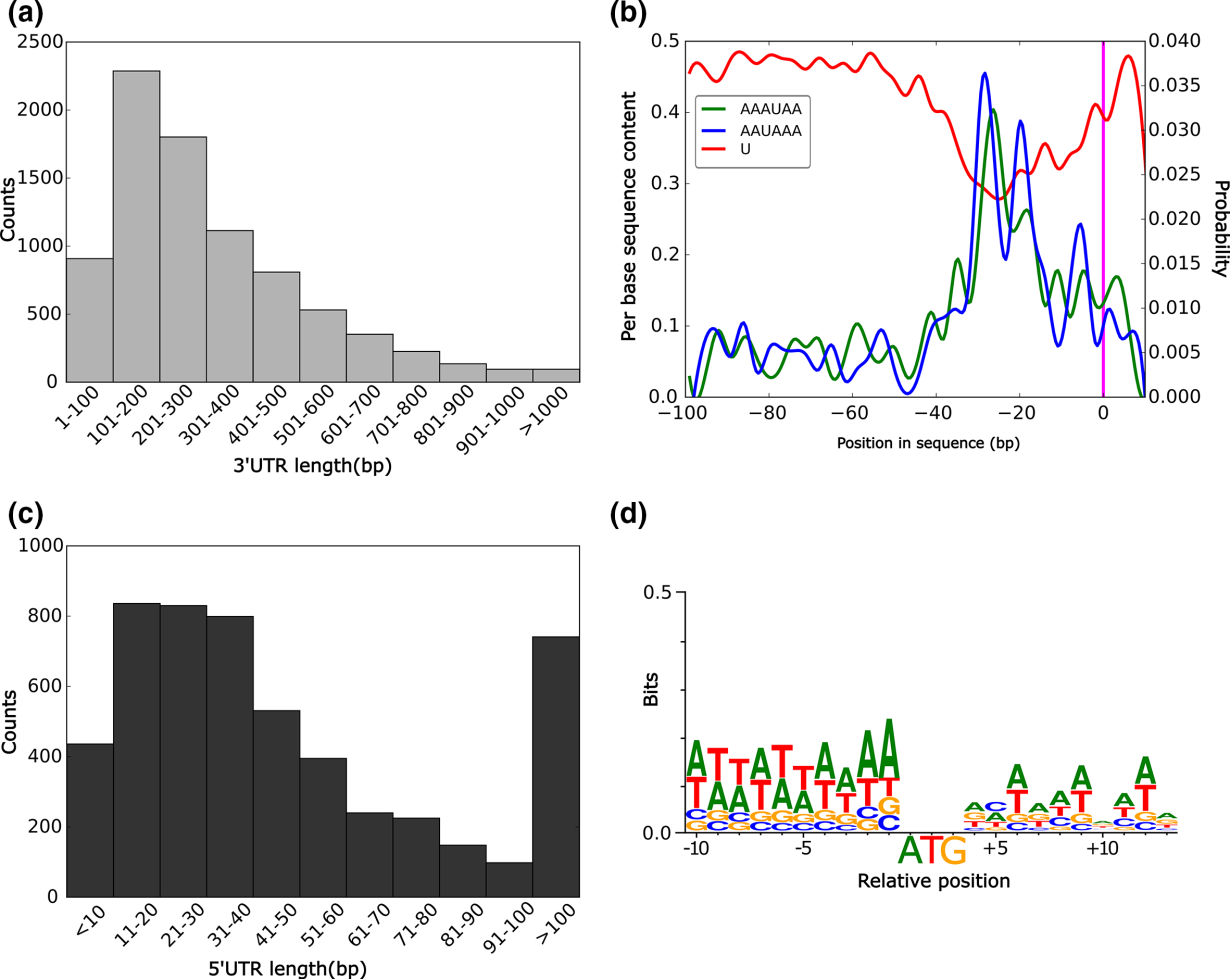
UTR characteristics in the genome of *Monocercomonoides exilis*. (a) 3′ UTR length distribution based on all annotated UTRs; (b) Single-nucleotide scan from positions −100 to +10 in the 3′ UTR upstream and downstream region. The occurrence probability of the two identified polyadenylation signals is represented on the second axis, and the average content of uridine bases is represented on the first axis. The pink line marks the position of the cleavage site; (c) 5′ UTR length distribution based on all annotated UTRs; (**d)** A sequence logo showing the conservation of the bases around the start codon based on 632 sequences. Larger letters indicate higher frequency of the bases at that location.

Using the ONT transcriptome reads, we managed to predict 3′UTR sequences for 8354 genes and 5′UTR sequences for 5279 genes. The sequences of both 5′ and 3′UTRs may not be fully complete as not all transcripts from ONT transcriptome used for prediction polishing could be classified as full-length. We identified 552 introns in the annotated 5′UTRs and only 218 introns in the annotated 3′UTR sequences. The average length of the 3′UTR sequences was 312 bp (Table 1) and the size distribution shows that most of the predicted UTRs are below 500 bp (Fig. 4a). The 3′UTRs seem to be AT-rich with a GC content of 26.13 %. To identify motifs for polyadenylation, we searched the last 100 bp using STREME [31] in a subset of 710 full length 3′ UTR sequences (see Methods). We identified two motifs, AAAUAA and AAUAAA, located between 20 and 30 bp from the cleavage site (Fig. 4b), which could serve as polyadenylation signals in *M. exilis*. These motifs were flanked by U-rich regions (Fig. 4b). Moreover, most components for signal recognition and polyadenylation were identified in the genome of *M. exilis* (Table S2).

Regarding 5′ UTRs, the situation was less clear. The average size of the 5′UTR is 62 bp, but size distribution shows a broader range with more than 700 UTR sequences displaying sizes larger than 100 bp (Fig. 4c). We characterised the Kozak consensus sequence by summarizing 10 bp upstream and downstream of the start codon on a subset of 632 full-length UTR sequences using Weblogo [33] (Fig. 4d). We noticed that the putative Kozak consensus sequence located upstream of the start codon is AT-rich, but no clear motif can be drawn from the logo.

### Mitochondrial proteins

We searched all 4665 newly predicted or corrected proteins for homologues of nuclear genome-encoded proteins typically associated with mitochondria or MROs in other eukaryotes. In the first step, we searched for mitochondrion protein import and maturation machinery, considered as one of the most conserved mitochondrial features. HMM homology searcher resulted in 28 candidates but only six had any homolog in the Mitominer database. Two of the candidates are very long, had no significant hits from NCBI (MONOS_2792 and MONOS_3516fu3517) and their mitominer hits have been shown to be also cytosolic and nuclear proteins (Table S3). For three other candidates (MONOS_14890, MONOS_18199 and MONOS_18387) phylogenies showed no relationship to any known mitochondrial proteins (Fig. S1). The nature of the last candidate MONOS_10855, was the most difficult to determine. This protein had significant hits from Pam18 mitochondrial import motor protein. However, phylogenetic analysis did not show that the protein is a clear homolog of Pam18, due to low branch supports (Fig S2). Moreover, the protein structure predicted by AlphaFold [56] was very different from the published/predicted structures of Pam18 proteins. MONOS_10855 is apparently a DnaJ protein other than Pam18, as DnaJ domains are found in many other types of proteins.

The homology-based searches were complemented by an extensive search for putative homologues of known mitochondrial proteins using a pipeline based on the Mitominer database [34], enriched with identified mitochondrial proteins of diverse anaerobic eukaryotes with MROs (see Methods). As already shown for *M. exilis*, the specificity of the pipeline in organisms with divergent mitochondrion is low [5]. In our case we recovered 326 candidates. Many of the selected candidates were annotated as proteins that are obviously not mitochondrial, but we recovered also several suspicious candidates (e.g. MONOS_5671 malonyl-CoA:pyruvate_transcarboxylase, MONOS_14754fu14870 putative nicotinamide nucleotide transhydrogenase, or MONOS_17803 putative cytosolic Fe-S cluster assembly factor NARFL). However, all those candidates lack the targeting signal and were previously considered and argued to be cytosolic [5]. None of the newly predicted proteins turned out to be promising candidates for mitochondrial proteins.

As an alternative to homology searches, we have also inquired for several types of signature sequences typical of mitochondrion-targeted proteins. The matrix proteins of mitochondria and MROs are expected to contain conserved N-terminal targeting signals required for targeted import into the organelles [57]. However, as we previously showed for *M. exilis*, prediction tools recognize almost 1 % of proteins to contain targeting signals despite the lack of mitochondrion [5]. Here we identified 24 candidates with predicted localization signal (0.5 %) out of 4665 analysed proteins and based on homology searches all candidates were identified as false positives (Table S3).

The outer mitochondrial membranes accommodate two special classes of proteins, β-barrel and tail anchored (TA) proteins, which use specific C-terminal signals [58–60]. We have identified seven candidate TA proteins (Table S3), with four of them bearing homologs in the Mitominer database, mainly as components of endomembrane trafficking system but not functioning in mitochondria. Another two proteins have been automatically recognised as β-barrel outer membrane proteins (MBOMPs) (Table S3). However, based on homology search, both have been annotated as cytosolic proteins and, upon manual evaluation, both have been identified as false positives (MONOS_2699 encodes clathrin heavy chain, which is a membrane protein involved in intracellular vesicle formation, while MONOS_10534 encodes spicing factor Prp8 and is too short to form a proper β-barrel channel).

## Discussion

Long-read sequencing (Pacific Biosystems and Oxford Nanopore Technologies, ONT) has been used for *de novo* sequencing or re-sequencing of several protist genomes in order to achieve contiguous genome assemblies [8, 10, 61–63]. Here we present a significantly improved draft genome of *Monocercomonoides exilis* strain PA203 after inclusion of ONT data. The *M. exilis* ONT genome assembly described in this study (NCBI accession number LSRY00000000, version 2) is around 82 Mbp in size, composes of 101 contigs (N50 value 1 379 369 bp) and it substitutes the 454 genome assembly version 2019-07-27 (https://giardiadb.org/giardiadb/app/record/dataset/DS_3a6ccbfbcf) and the genome assembly from NCBI with the accession number GCA_001643675.1 [5]. The assembly contains ten full-length chromosomes as well as 65 contigs with one telomeric end. Assuming that each contig containing one telomeric end represents one end of a chromosome, one may suggest that *M. exilis* bears anywhere from 40 to 50 chromosomes. Previous estimations [7] based on the genome sequence, as well as fluorescence *in situ* hybridization (FISH), suggested that *M. exilis* genome is organized in only 6–7 chromosomes, apparently a substantial underestimate. FISH is far from an accurate technique for estimating the number of chromosomes, and lower counts of telomeric signals using FISH have also been observed in other protists [64], possibly caused by poor labelling efficiency and probe accessibility to the telomeric regions, as well as potential overlap of multiple signals. While the number of chromosomes estimated from our assembly is higher than that of other metamonads like *Giardia* or *Trichomonas* [65–67], it is not unusual among protists [68].

The ONT assembly is approximately 8 Mbp longer than the previously published 454 assembly. The increase in size is partially caused by the resolution of repetitive elements which do not collapse anymore. Increased assembly lengths have also been achieved in other re-sequenced genomes [9, 63]. Although the genome is larger, the genome completeness, estimated using CEGMA [30] remained unchanged. The new genome assembly includes 1829 new gene models, which were not predicted in the 454 assembly, although for most of them, a major part of their nucleotide sequence was present in the 454 assembly. A total of 1637 of the newly predicted gene models are hypothetical proteins and none of the remaining bring new structural or metabolic functions.

Repeat analysis using RepeatModeler suggested that around 45.97 % of the new genome assembly is represented by various types of repeats (Table 2), higher than the initially reported 37–38 % [5, 16] and consistent with the size increase of the assembly. In metamonads, the percentage of repetitive elements varies from 4 % in *Carpediemonas frisia* up to approximately 67 % in *Trichomonas vaginalis* [16], placing the genome of *M. exilis* on an average measure of repetitive elements-content. Yet, the relative composition of repetitive elements is very different to other metamonads, containing the highest percentage of unclassified repeats (Table 2) [16]. While these could represent some new types of repeats, we hypothesise that some of the unclassified repeats may be misidentified and may represent highly expanded protein families in the genome of *M. exilis,* such as protein tyrosine kinases, which tend to overlap with unclassified repeats (Fig. 1).

Besides the improvement in contiguity, several sequence errors were corrected in the ONT assembly. The published 454 assembly was based on 454 sequencing reads which are prone to errors in homopolymeric regions [69]. As ONT sequencing is also prone to such errors [13, 70] mapping of newly obtained Illumina reads was used to manually investigate and resolve each suspicious case. In this procedure, we found and corrected altogether three frameshifts, which were not corrected by Pilon or Nanopolish. Interestingly, the same procedure revealed around 120 frameshifts in the original 454 assembly which remained unnoticed and affected gene predictions. As expected, these frameshifts were mainly in homopolymeric regions and could have been caused by the slightly lower genome coverage of the 454 reads [5, 7]. Our results show that even careful correction of the ONT assembly with Nanopolish and Pilon fails to fully correct all positions. Our hypothesis is that certain parts of the genome, present in multiple copies, are not polished at the same level as the unique parts during Nanopolish correction. This in turn affects how the short reads map back to the genome with BWA [28], leaving some parts of the genome only with secondary alignments. As Pilon requires ‘the single best hit’ or ‘random selection among equal best alignments’ [19], the lower scoring alignments will be ignored, causing Pilon to correct only the areas with the best hit alignments. Indeed, we noticed such scenarios during our manual investigation and overcame this issue by looking into secondary alignments or mapping the Illumina reads one contig at the time. It is possible that multiple iterations of Nanopolish may mitigate this issue as it has been done in other assemblies [9].

The overall improvements of the genome assembly had a big impact on the gene prediction quality. The published 454 genome assembly contained more than a thousand manually annotated and curated gene models [7], but also approximately 500 gene models were partial mainly due to assembly fragmentation. Using a combination of Augustus, EVM, RATT and PASA we successfully managed to transfer most genome annotations (Table 1) while maintaining the locus tags, thus any previous gene annotation may be easily identifiable in the ONT assembly. Automatic methods failed to transfer around 1200 gene models, which were subsequently transferred manually. To improve the gene prediction, we also used long-read transcriptomic reads from ONT sequencing. Long-read transcriptomic data has been shown to significantly improve gene predictions in several organisms [71–73], but incorporating such data in existing annotations is challenging due to the lack of ready-to-use pipelines. Recently developed pipelines meant to use long-read transcriptomic data like LoReAn [74], do *de novo* predictions that are further improved by short-read and long-read transcriptomic data, yet the pipeline is unable to use pre-existing annotations. We overcame this issue using a step-by-step approach with the PASA pipeline. This method managed to add UTR annotations and improve overall prediction accuracy, as reflected by the increased prediction completeness after each run (Fig. 3). As the number of gene models expanded, so did the introns associated with them, yet the intron density remained virtually unchanged (Table 1).

The UTR annotations were not transferred from the previous assembly due to their short size, and manual investigation showed that some contain fused parts of other genes. This was most probably caused by usage of unoriented Illumina transcriptomic data in the previous annotation [5]. In the new predictions, UTRs were annotated *de novo* using the long-read ONT transcriptome. The average length of the 3′UTR increased, but the average length of the 5′UTR annotations decreased (Table 1). We identified more introns in the 5′ UTR compared to the 3′UTR sequences. A similar pattern has also been observed in the human genome [75]. Some studies have shown that the presence of introns in the 5′UTR may upregulate gene expression [76], while the presence of introns in the 3′UTR may have a negative effect on the expression levels [77].

UTRs may also contain various motifs and structures such as alternative start codons, hairpins, ribosomal entry sites, polyadenylation sites, micro-RNA binding sites, all of which may affect the stability and translation of certain mRNAs [78, 79]. One important regulatory element in the 5′ UTR is the Kozak consensus sequence, a motif located upstream the start codon playing a role in translation initiation [80, 81]. We show that the putative Kozak consensus sequence in *M. exilis* is AT-rich, but a defined sequence motif was not revealed (Fig. 4d). Regardless, the region shares similarity with consensus sequences identified in other eukaryotes including protists [82–84], but differs from the GC-rich motif found in most vertebrates [80].

The polyadenylation signal is one of many regulatory elements present in 3′UTRs [85]. This signal is recognized by cleavage and polyadenylation specificity factor which in turn stimulates cleavage of the 3′end of the precursor mRNA and addition of the polyA tail by polyA polymerase [85, 86]. The polyadenylation signal in mammals has been identified as A(A/U)UAAA [87, 88] and is located between 10–30 bases upstream of the cleavage site, usually surrounded by U-rich elements. The polyadenylation motifs identified in *M. exilis* seem canonical (AAAUAA and AAUAAA), are located between 20 and 30 bp (Fig. 4b) upstream the 3′UTR end and surrounded by U-rich elements. This fact further suggests that *M. exilis* has canonical eukaryotic complexity, even though it lacks a mitochondrion. Regardless of its resemblance to the polyadenylation signals in metazoans and *Giardia intestinalis* (AGUAAA) [89, 90], it differs from *Trichomonas vaginalis* (UAAA) [91]. Apparently, the polyadenylation signal diverged in the lineage leading to trichomonads, yet it remainedfully functional [92].

The much-improved genomic draft was used to re-test the hypothesis of the amitochondriate status of the species. We have carefully inspected 4665 gene models, either newly predicted or modified, for the presence of putative nucleus-encoded mitochondrial proteins using homology-based and signature-sequence-based approaches. None of the searches has revealed any strong candidate to reject the current hypothesis, and so we continue to regard *M. exilis* to be an amitochondriate species.

## Supplementary Data

Supplementary material 1

Supplementary material 2

Supplementary material 3

Supplementary material 4
