# Supplementary material for: High quality genome assembly of the amitochondriate eukaryote Monocercomonoides exilis
Source: Microb Genom. 2021 Dec 24;7(12):000745. doi: 10.1099/mgen.0.000745 (PMC8767320; doi:10.1099/mgen.0.000745)
Supplement: Supplementary material 1 [file mgen-7-0745-s001.pdf]

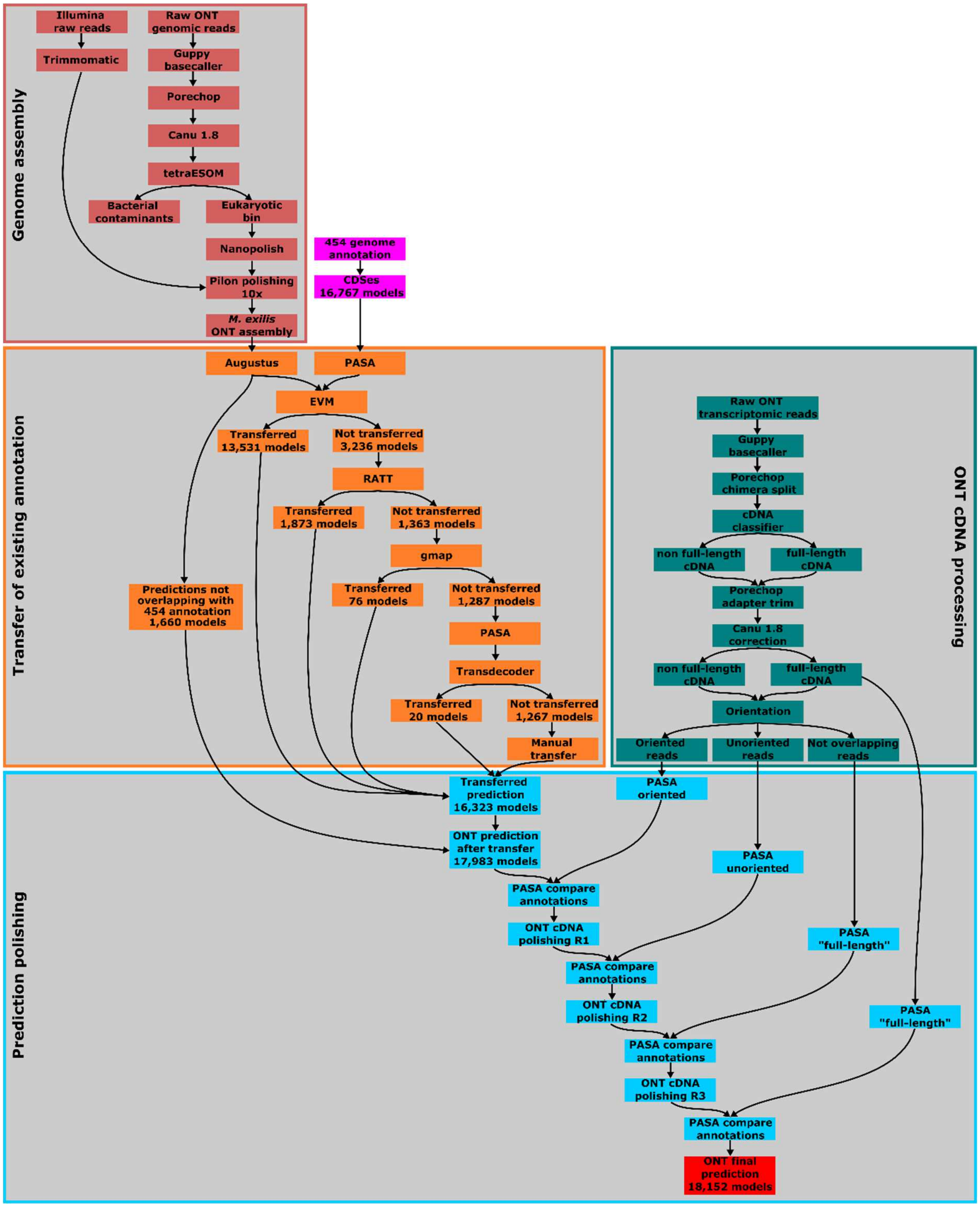

Figure S1. A schematic overview of the genome assembly, annotation transfer and annotation polishing. The number in the Pilon box represents the number of runs used for polishing the genome. The published data used to transfer the annotation is represented in pink boxes.

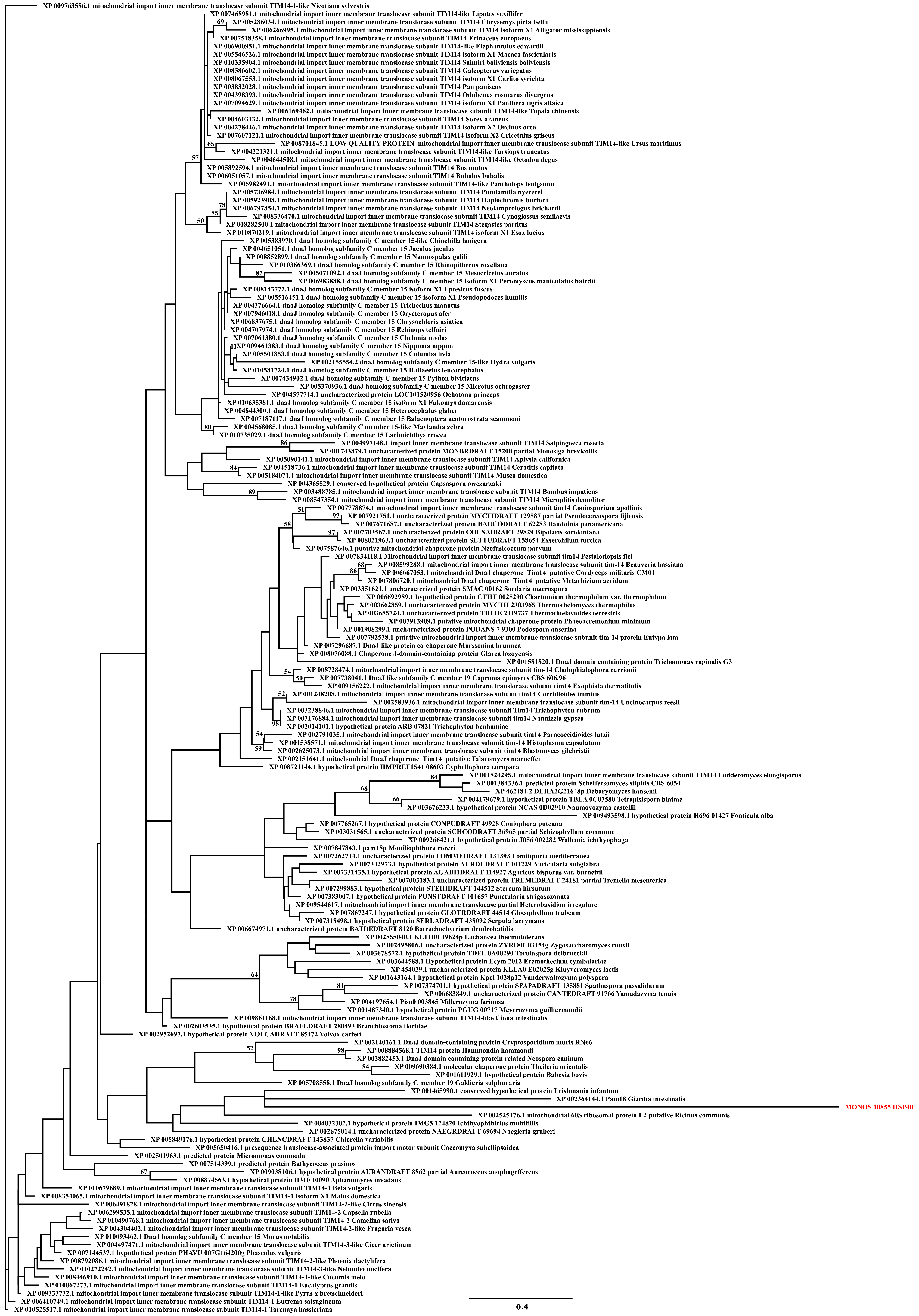

Figure S2: Maximum-likelihood tree constructed for MONOS_10855 gene. The tree was constructed using IQ-TREE. Values at the nodes represent non-parametric bootstraps support values (>50%).

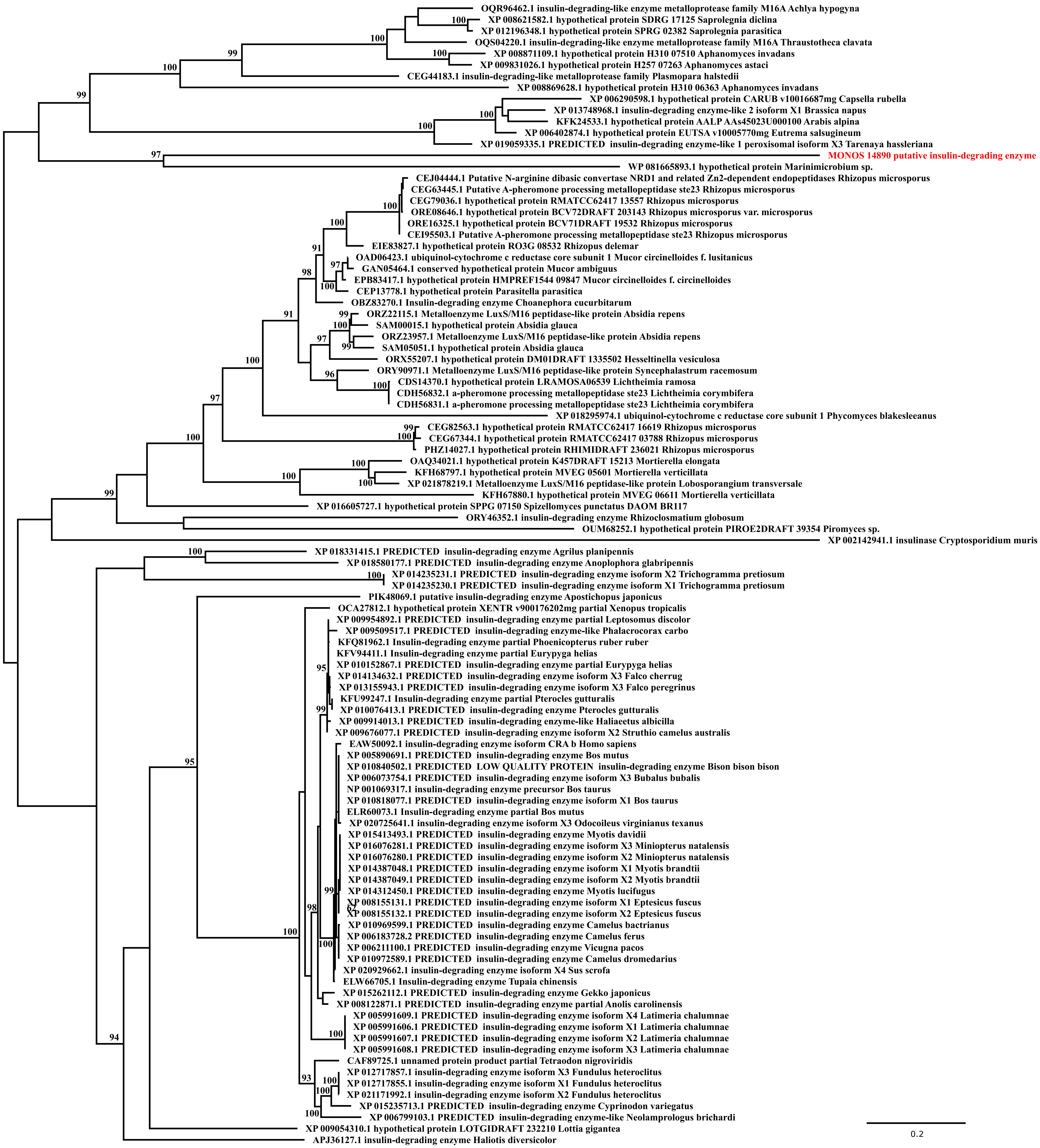

Figure S3: Maximum-likelihood tree constructed for MONOS_14890 gene. The tree was constructed using IQ-TREE. Values at the nodes represent Ultrafast bootstraps support values (>90%).

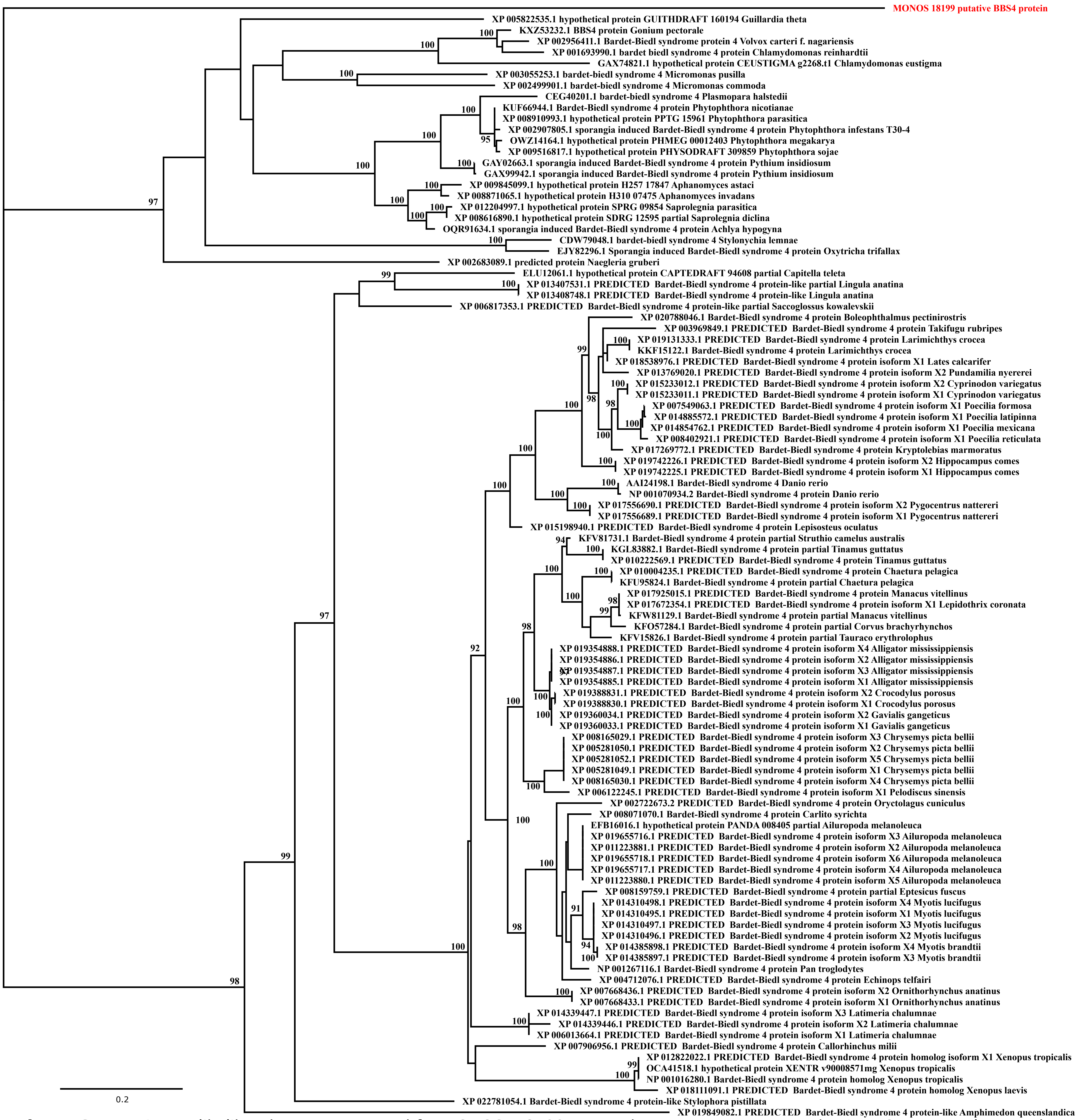

Figure S4: Maximum-likelihood tree constructed for MONOS_18199 gene. The tree was constructed using IQ-TREE. Values at the nodes represent Ultrafast bootstraps support values (>90%).

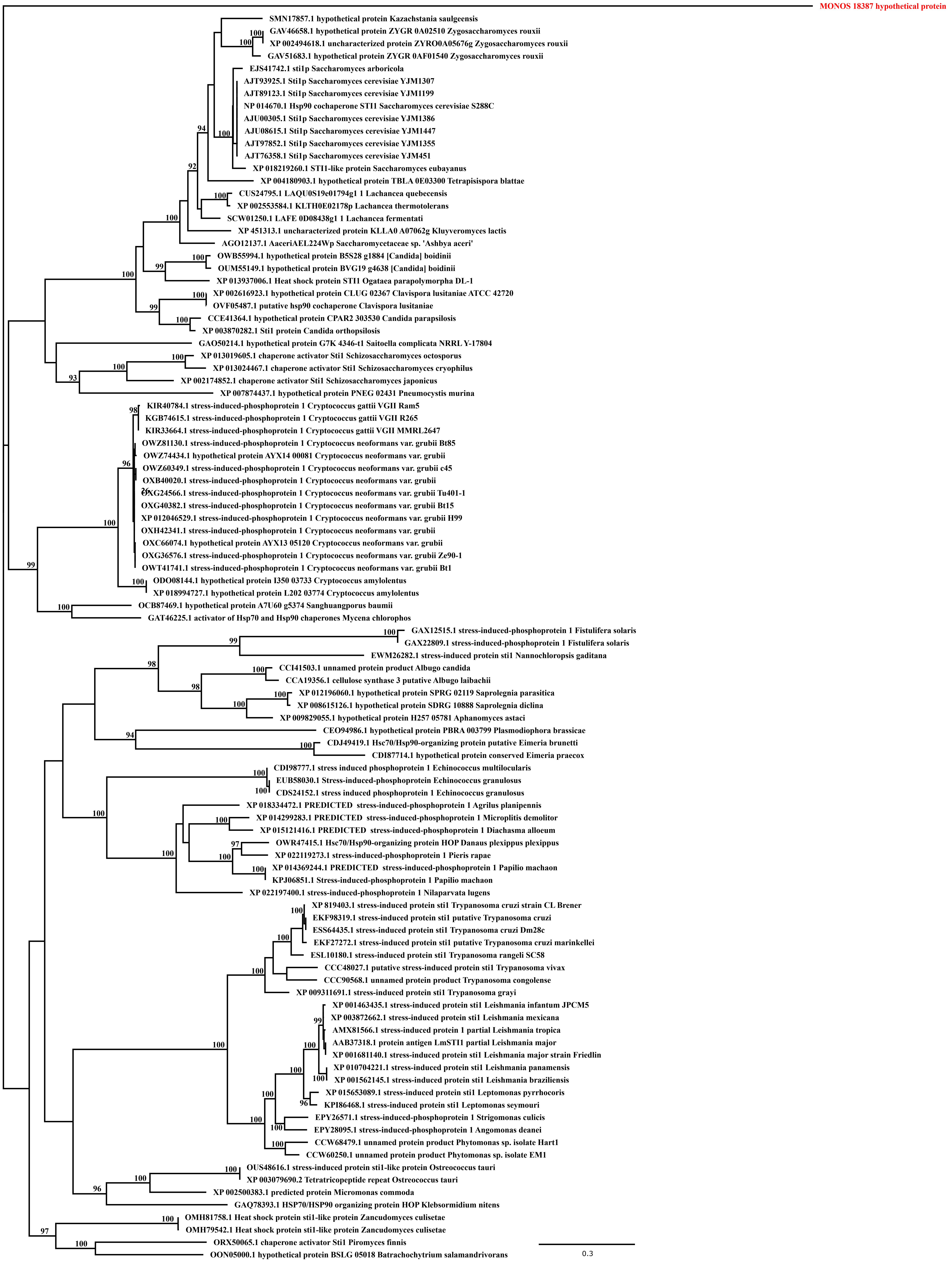

Figure S5: Maximum-likelihood tree constructed for MONOS_18387 gene. The tree was constructed using IQ-TREE. Values at the nodes represent Ultrafast bootstraps support values (>90%).
